# The Function of Autophagy in Neurodegenerative Diseases

**DOI:** 10.3390/ijms161125990

**Published:** 2015-11-09

**Authors:** Yoshimitsu Kiriyama, Hiromi Nochi

**Affiliations:** Kagawa School of Pharmaceutical Sciences, Tokushima Bunri University, Shido 1314-1, Sanuki, Kagawa 769-2193, Japan; kiriyamay@kph.bunri-u.ac.jp

**Keywords:** autophagy, mitophagy, Alzheimer’s disease, Parkinson’s disease, Huntington’s disease, ALS, SENDA

## Abstract

Macroautophagy, hereafter referred to as autophagy, is a bulk degradation process performed by lysosomes in which aggregated and altered proteins as well as dysfunctional organelles are decomposed. Autophagy is a basic cellular process that maintains homeostasis and is crucial for postmitotic neurons. Thus, impaired autophagic processes in neurons lead to improper homeostasis and neurodegeneration. Recent studies have suggested that impairments of the autophagic process are associated with several neurodegenerative diseases, such as Alzheimer’s disease, Parkinson’s disease, Huntington’s disease, amyotrophic lateral sclerosis, and static encephalopathy of childhood with neurodegeneration in adulthood. In this review, we focus on the recent findings regarding the autophagic process and the involvement of autophagy in neurodegenerative diseases.

## 1. Introduction

Autophagy is a catabolic process that degrades the cytosolic components, such as proteins and organelles, by transporting them to the lysosome, and the process of autophagy is regulated by autophagy-related (ATG) proteins [1,2,3,4]. It has been shown that autophagy is activated by starvation, including a deficiency in amino acids or glucose [5,6]. In addition, the functions of autophagy are to digest defective or aggregated proteins, to clear damaged organelles, differentiation, and development [7,8,9]. There are three different types of autophagy: macroautophagy, microautophagy, and chaperone-mediated autophagy. Macroautophagy, hereafter referred to as autophagy, is the process of bulk degradation of proteins and organelles via the formation of an autophagosome, a double-membraned vesicle that isolates proteins and organelles. To degrade the cytosolic components, the autophagosome fuses with a lysosome to form an autolysosome [9]. Autophagy plays an important role in cellular quality control in neurons because the denatured or aggregated proteins and the dysfunctional organelles are not reduced by cell division in neurons. Moreover, the functional impairment of autophagy leads to neurodegenerative disorders [10,11]. In this review, we focus on the recent advances in understanding the physiological and pathophysiological roles of autophagy that are related to the mechanisms of neurodegeneration.

## 2. Autophagy Machinery

The processes in autophagy include the initiation, elongation and closure, and maturation stages. The depletion of nutrients, such as amino acids or glucose, induces autophagy. However, recent studies have shown that autophagy can be induced by diverse stimulations, such as denatured or aggregated proteins, organelle damage, reactive oxygen species, hypoxia, and stress [12].

**Figure 1:**
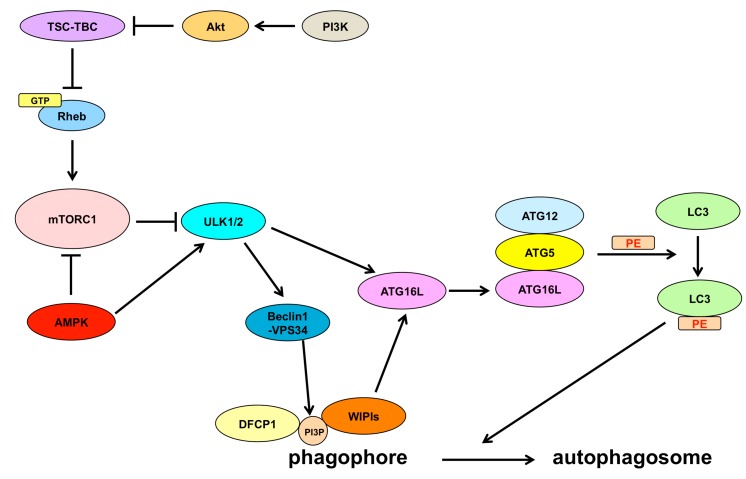
Mechanisms of autophagy. The PI3K-Akt pathway inhibits the TSC-TBC complex, which inhibits Rheb; therefore, inhibition of the TSC-TBC complex results in the activation of Rheb when Rheb is converted from the GDP-bounded form to the GTP-bounded form. GTP-loaded Rheb activates mTORC1 and inhibits the ULK1/2 complex, leading to the inhibition of the autophagic flux. On the other hand, AMPK inhibits mTORC1 and activates the ULK1/2 complex. Stresses, such as the accumulation of denatured or aggregated proteins, damaged organelles, and reactive oxygen species, inhibit mTORC1 or activate the ULK1/2 complex, resulting in the induction of autophagy. The ULK1/2 complex activates the Beclin1-VPS34 complex and ATG16L. The activated Beclin1-VPS34 complex functions as a class III PI3K to produce PI3P. WIPI proteins and DFCP1 are recruited to PI3P to generate a phagophore. WIPI proteins also activate ATG16L. ATG16L forms the ATG12–ATG5–ATG16L complex with ATG12 and ATG5. The ATG12–ATG5–ATG16L complex conjugates PE to LC3 to expand the phagophore and form an autophagosome. TSC: tuberous sclerosis; TBC: Tre2-Bub2-Cdc16; Rheb: Ras homolog enriched in brain; mTORC1: the mammalian (or mechanistic) target of rapamycin complex 1; ULK1/2: Unc-51-like kinase 1/2; PI3P: phosphatidylinositol 3-phosphate; WIPI: WD-repeat protein interacting with phosphoinositides; DFCP1: double FYVE domain-containing protein 1; PE: phosphatidylethanolamine.

As shown in Figure 1, the mammalian (or mechanistic) target of rapamycin complex 1 (mTORC1) has been shown to be the key regulator controlling the initiation step of autophagy [13]. mTORC1 is activated by guanosine-5′-triphosphate (GTP)-loaded Ras homolog enriched in brain (Rheb), which is on the lysosome membrane. Rheb is a small GTPase; GTP-loaded Rheb is the active form [14]. The TSC–TBC complex, composed of tuberous sclerosis 1 (TSC1), TSC2, and Tre2-Bub2-Cdc16 1 domain family member 7 (TBC1D7), negatively regulates Rheb. TSC2 in the TSC–TBC complex possesses GAP activity for Rheb and inhibits the activity of Rheb by changing GTP to GDP. The phosphoinositide 3-kinase (PI3K)–v-akt murine thymoma viral oncogene homolog 1 (Akt) pathway, by the mediation of growth factors, such as insulin-like growth factor 1 (IGF-1) and epidermal growth factor (EGF), phosphorylates and inhibits the TSC–TBC complex, and then GTP-loaded Rheb is formed and mTORC1 is activated [15]. Activated mTORC1 negatively controls autophagy by blocking the Unc-51-like kinase (ULK)1/2 complex, an initial inducer of autophagy that includes ULK1/2, ATG13, and focal adhesion kinase family interacting protein of 200 kD (FIP200) [16,17]. Therefore, the inhibition of mTORC1 activates the ULK1/2 complex and is the induction of the autophagic process. On the other hand, AMP-activated protein kinase (AMPK) inhibits mTORC1 and activates the ULK1/2 complex [18]. The activated ULK1/2 autophosphorylates and phosphorylates ATG13 and FIP200 in the ULK1/2 complex [16,17,19,20]. The ULK1/2 complex activates the Beclin1-vacuolar protein sorting 34 (VPS34) complex, which contains Beclin1, VPS34, and ATG14L [21], by phosphorylating Beclin1 [22]. The activated Beclin1-VPS34 complex functions as a class III PI3K to produce phosphatidylinositol 3-phosphate (PI3P), and PI3P recruits WD-repeat protein interacting with phosphoinositides (WIPI) proteins and double Fab1, YGLO23, Vps27, and EEA1 (FYVE) domain-containing protein 1 (DFCP1). WIPI proteins and DFCP1 are PI3P binding proteins. Recruited WIPI proteins and DFCP1 play a role in generating a phagophore (isolation membrane), a premature membrane structure of an autophagosome [23,24,25,26]. WIPI2, one of the WIPI proteins, recruits ATG16L to a phagophore [23]. Moreover, FIP200 in the ULK1/2 complex also interacts with ATG16L [27,28]. ATG16L binds ATG5 and ATG12 to form the ATG12–ATG5–ATG16L complex, and the ATG12–ATG5–ATG16 complex conjugates phosphatidylethanolamine (PE) to microtubule-associated protein 1 light chain 3 beta (LC3B) [29]. The PE-conjugated LC3B functions to elongate and form the autophagosome. Moreover, LC3B carries denatured and aggregated proteins or damaged organelles into an autophagosome [30]. LC3B is the mammalian ortholog of yeast ATG8. The mammalian ortholog of yeast ATG8 contains six proteins, which are divided into the LC3 subfamily and the γ-aminobutyric-acid-type-A receptor-associated protein (GABARAP) subfamily. The LC3 subfamily comprises LC3 alpha (LC3A), LC3B, and LC3C. The GABARAP subfamily comprises GABARAP, GABARAP-like 1 (GABARAPL1), and GABARAP-like 2 (GABARAPL2). Both the LC3 subfamily members and the GABARAP subfamily members function in the formation of the autophagosome [30,31,32,33,34]. The autophagosome membrane is derived from a variety of sources, including the endoplasmic reticulum (ER) [35,36], mitochondrial membrane [37], plasma membrane [38], Golgi [39], and recycling endosomes [40]. ER is the major source of the autophagosome membrane [36] and physically connects with the isolation membrane [35,36,41,42]. The Ω-like shaped isolation membrane that contains PI3P and DFCP1, called the omegasome, arises from ER, develops into the autophagosome [35,36], and is held between two sheets of the ER membrane [36,43]. These findings indicate that the isolation membrane is generated from ER and ER guides the elongation of the isolation membrane. ULK1, which functions at the initiation step of autophagy, is recruited to the ER membrane to promote the formation of the isolation membrane [24]. Thus, the structure and function of the ER play a crucial role in the early stages of autophagy. When the formation of the autophagosome is complete, the autophagosome fuses with the lysosome to become the autolysosome. The enzymes in the lysosome degrade the substrates, which are brought by the autophagosomes. The fusion of an autophagosome with a lysosome is mediated by soluble *N*-ethylmaleimide-sensitive factor attachment protein receptors (SNAREs) [44,45,46], Rab7 [47,48], UV radiation resistance-associated gene protein (UVRAG) [49], the homotypic fusion and protein sorting (HOPS) complex [50], LC3 [51], and GABARAPs [52]. Although mTORC1 inhibits the initiation of autophagy by blocking the activity of the ULK1/2 complex, mTORC1 also inhibits the fusion of an autophagosome and a lysosome by phosphorylating UVRAG [53]. This indicates that mTORC1 negatively controls the process of autophagy from the initiation stage to the maturation stage.

Recent studies have shown that dysfunction of TAR DNA-binding protein 43 kDa (TDP-43) or fused in sarcoma (FUS) leads to the impairment of autophagy [54,55]. TDP-43 and FUS are RNA-binding proteins that function in various RNA processing steps, such as stabilizing mRNA and splicing pre-mRNA [56]. Loss of function of TDP-43 destabilizes ATG7 mRNA and causes a reduction in its level, leading to the impairment of autophagy [54] because ATG7 plays an important role in the formation of autophagosomes [57,58]. On the other hand, mutated FUS restrains autophagy by inhibiting autophagosome formation, and Rab1 recovers this autophagosome formation [55]. Rab1 regulates autophagosome formation [59]. It was also indicated that mutated FUS may inhibit the activity of Rab1 because mutation of FUS does not change the expression level of Rab1 [55]. FUS may play a crucial role in the regulation of Rab1 activity by affecting the stabilization of GTPase regulator mRNAs, because FUS binds to these [60]. These findings indicate that autophagy may be controlled by the stabilization of the mRNAs of proteins that are associated with autophagy or membrane trafficking.

## 3. Neurodegeneration and Autophagy

Aggregated and ubiquitinated proteins cause synaptic impairment, damage to organelles, and cell death in the central nervous system. Many types of neurodegenerative diseases are accompanied by the accumulation of aggregated and ubiquitinated proteins. Autophagy is involved in the degradation and removal of aggregated proteins, and the inhibition of constitutive autophagy leads to neurodegeneration in the central nervous system [10,11]. Autophagic flux is controlled by the balance between autophagosome formation and autophagic degradation, the impairment of which causes neuronal cell death [61,62]. The accumulation of autophagosomes in neurons is associated with neurodegenerative diseases, such as Alzheimer’s disease [63,64], Parkinson’s disease [65], and Huntington’s disease [66]. Such accumulation indicates an imbalance between autophagosome formation and autophagic degradation. Excessive autophagy can lead to excessive degradation of cytosolic components and neuronal cell death [67,68,69]. Inhibition of the transport of autophagosomes by the disassembly of microtubules or the deletion of histone deacetylase (HDAC6), a tubulin deacetylase, leads to inhibition of the fusion of an autophagosome with a lysosome and is associated with neurodegenerative diseases [70,71,72]. Furthermore, disturbances of endosomal trafficking are associated with neurodegenerative diseases [73]. The multivesicular body (MVB) plays a crucial role in transporting membrane proteins to the lysosome for degradation. The formation of MVBs is regulated by the endosomal sorting complexes required for transport (ESCRT) [74]. Impairment of the ESCRT-III complex compromises the formation of MVBs and leads to autophagosome accumulation and neurodegeneration [75]. The reduction of lysosomal acidification or decreased activity of lysosomal hydrolases leads to the inhibition of autophagic degradation and neurodegenerative diseases [9]. Thus, an imbalance of autophagic flux is strongly associated with neurodegeneration. Recent studies have shown that mutations in autophagy-related genes and the altered autophagic flux cause neurodegenerative diseases such as Alzheimer’s disease, Parkinson’s disease, Huntington’s disease, amyotrophic lateral sclerosis (ALS), and static encephalopathy of childhood with neurodegeneration in adulthood (SENDA).

### 3.1. Alzheimer’s Disease

Alzheimer’s disease is the most common progressive neurodegenerative disorder and is characterized by dementia and morphological changes in the brain. The pathology of brains from individuals with Alzheimer’s disease indicates the amyloid plaques composed of accumulated amyloid-β (Aβ) peptides and the neurofibrillary tangles with tau [76]. Amyloid precursor protein (APP) is a transmembrane protein that is processed by β-secretase and γ-secretase, and the cleavage of APP by these two enzymes produces Aβ peptide [77,78]. The extracellular amyloid plaques are possibly generated by Aβ peptides, which are produced inside of the cell. The autophagosome has γ-secretase activity and is the place in which Aβ peptides are generated and accumulate in neurons from mouse models of Alzheimer’s disease [64,79,80]. The excessive accumulation of autophagosomes in dystrophic neurites was observed in the brains of patients with Alzheimer’s disease [63,64]. This indicates that there is a disruption in the degradation of cytosolic components in autolysosomes. Presenilin 1 is included in the γ-secretase complex [81] and plays an important role in the maturation of V-ATPase, which is necessary for the acidification of lysosomes [82]. Dysfunction of presenilin 1, which is associated with familial Alzheimer’s disease, causes the impairment of the acidification of lysosomes. The impairment of lysosomal acidification results in the inhibition of proteolysis in autolysosomes and the disruption of autolysosome formation, leading to the accumulation of autophagosomes and Aβ peptides in autophagosomes.

Beclin1, which is necessary for the initiation of autophagy, has been shown to be reduced in the brains of patients with Alzheimer’s disease, and the reduction of beclin1 leads to decreased autophagy, the accumulation of Aβ peptides, and neurodegeneration in mouse models of Alzheimer’s disease [83,84]. Moreover, the induction of autophagy via the inhibition of the mTOR signaling pathway by rapamycin reduces the levels of Aβ peptides and improves cognitive impairments in mouse models of Alzheimer’s disease [85,86,87]. These findings suggest that autophagy plays a crucial role in Alzheimer’s disease, and controlling autophagy in neurons might be a potential treatment for Alzheimer’s disease.

### 3.2. Parkinson’s Disease

**Figure 2:**
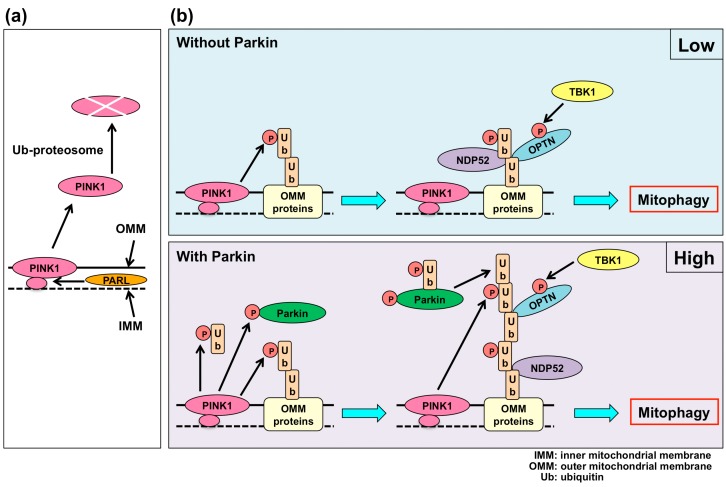
Mitophagy with PINK1 and Parkin. (**a**) When mitochondria are healthy, PINK1 is processed by PARL in mitochondria, and the processed PINK1 is degraded by the ubiquitin-proteasome system; and (**b**) When mitochondria are depolarized, PINK1 stays on the OMM. PINK1 phosphorylates ubiquitin, which is originally linked to an OMM protein. Without Parkin, NDP52 and OPTN can bind a phospho-ubiquitin-linked OMM protein and induce mitophagy. Autophagic flux is low under these conditions. With Parkin, PINK1 phosphorylates ubiquitins and Parkin. Parkin is activated by phosphorylation and binding with phosphorylated-ubiquitin. The activated Parkin adds ubiquitins to a phospho-ubiquitin-linked OMM protein, and PINK1 phosphorylates ubiquitins in a polyubiquitinated OMM protein. NDP52 and OPTN bind the polyubiquitinated OMM protein and induce mitophagy. Autophagic flux is high under these conditions. The phosphorylation of OPTN by TBK1 enhances the binding ability of OPTN to ubiquitinated-OMM and LC3. PINK1: phosphatase and tensin homolog (PTEN) induced putative kinase 1; PARL: presenilin-associated rhomboid-like; IMM: inner mitochondrial membrane; OMM: outer mitochondrial membrane; NDP52: nuclear dot protein 52 kDa; OPTN: optineurin; TBK1: TANK-binding kinase 1; P: phosphorylation.

Parkinson’s disease is the second-most common neurodegenerative disease after Alzheimer’s disease. Patients with Parkinson’s disease show tremors, rigidity (muscle stiffness), akinesia (loss or impairment of voluntary movements), bradykinesia (slowness of movement), and postural instability [88]. Parkinson’s disease is caused by the selective death of dopaminergic neurons in the substantia nigra, leading to a loss of dopamine in the striatum [88,89]. Mutations in specific genes have been identified in familial Parkinson’s disease, although approximately 90% of Parkinson’s disease is sporadic [90]. Mutations in phosphatase and tensin homolog (PTEN)-induced putative kinase 1 (PINK1), which is encoded by the *PARK6* gene, were identified in cases with early-onset Parkinson’s disease [91,92]. On the other hand, mutations in Parkin, which is encoded by the *PARK2* gene, were also identified in early-onset Parkinson’s disease [93,94,95]. Mutations in PINK1 or Parkin are the leading cause of parkinsonism [96]. PINK1 contains a mitochondrial targeting sequence and a serine/threonine kinase domain [91]. PINK1 is processed by presenilin-associated rhomboid-like (PARL), a protease in mitochondria, under healthy mitochondria conditions [97,98], and the processed PINK1 is rapidly degraded by the ubiquitin-proteasome system [99] (Figure 2a). In depolarized mitochondria, the processing of PINK1 by PARL is inhibited and PINK1 accumulates on the outer mitochondrial membrane (OMM) [97,98] (Figure 2b).

PINK1, on the depolarized mitochondria, autophosphorylates and recruits Parkin to damaged mitochondria [100]. Moreover, PINK1 phosphorylates ubiquitin and Parkin, leading to the activation of Parkin [101,102,103]. Parkin is an enzyme3 (E3) ubiquitin ligase, and Parkin-dependent ubiquitination sites are identified in many OMM proteins [104]. It was suggested that OMM proteins ubiquitinated by Parkin recruit p62, which is the ubiquitin-binding autophagic adaptor protein, and p62 mediates the binding between ubiquitinated OMM proteins and LC3 to be integrated in an autophagosome for the degradation of damaged mitochondria by autophagy (mitophagy) [105,106]. However, the role of p62 in mitophagy is controversial because p62 was not indispensable for mitophagy [107,108]. A recent study has shown that nuclear dot protein 52 kDa (NDP52) and optineurin (OPTN) among five ubiquitin-binding autophagic adaptor proteins—p62, Tax1 binding protein 1 (TAX1BP1), neighbor of BRCA1 gene 1 (NBR1), OPTN, and NDP52—were necessary for PINK1-Parkin-dependent mitophagy [109]. Although PINK1 initiates mitophagy in the absence of Parkin, mitophagy is significantly increased in the presence of Parkin (Figure 2b). PINK1 phosphorylates ubiquitin, which is originally linked to OMM proteins, and recruits OPTN and DPN52 to mitochondria [109]. The enhancement of mitophagy by Parkin may be caused by the addition of ubiquitins to the originally ubiquitinated OMM proteins by Parkin and the phosphorylation of ubiquitins in the polyubiquitinated OMM proteins by PINK1 [109,110]. The phosphorylation of OPTN by TANK-binding kinase 1 (TBK1) enhances the binding ability of OPTN to ubiquitinated OMM proteins and LC3 [111,112]. Furthermore, NDP52 and OPTN recruit autophagy-related proteins, such as ULK1, DFCP1, WIPI1, and LC3, to initiate autophagy [109]. Therefore, the dysfunction of PINK1 and Parkin in patients with Parkinson’s disease results in the impairment of mitophagy, which is regulated by NDP52 and OPTN.

### 3.3. Huntington’s Disease

**Figure 3:**
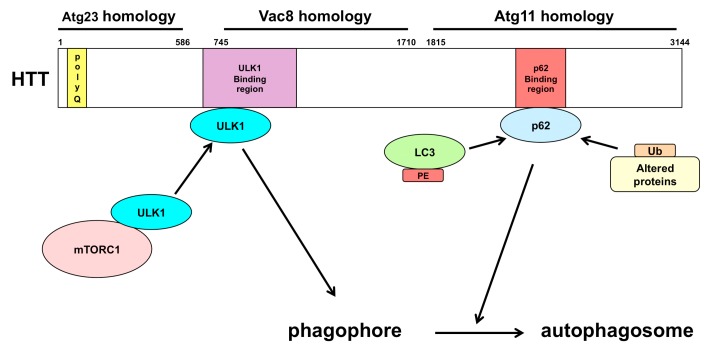
The structural similarity of HTT with yeast autophagic proteins (Atg23, Vac8, and Atg11) and the binding sites of ULK1 and p62 in HTT. HTT is similar to three yeast autophagic proteins (Atg23, Vac8, and Atg11). Moreover, HTT associates with ULK1 and p62. HTT binds ULK1 by competing with mTORC1 and may induce autophagy. HTT also binds to p62, which binds with a ubiquitinated substrate and LC3, and may carry them to an autophagosome. HTT: huntingtin; mTORC1: the mammalian (or mechanistic) target of rapamycin complex 1; ULK1: Unc-51-like kinase 1.

Huntington’s disease is an autosomal-dominant neurodegenerative disease caused by a cytosine-adenine-guanine (CAG) expansion encoding a polyglutamine (polyQ) at the N-terminus of huntingtin (HTT) and is characterized by motor dysfunctions, cognitive disability, and psychiatric disturbance [113,114]. Dysfunction of HTT results in neurodegeneration, indicating that HTT is essential for neurons [115,116]. HTT is a large and a flexible protein, and thus it can act as a multifunctional protein and play an important role in multiple cellular pathways by interacting with various proteins [117]. The polyQ region in HTT affects the structure of HTT and the interaction between HTT and other proteins, possibly leading to changes in cellular pathways [118,119]. HTT is similar to three different autophagic proteins in yeast: Atg23, Vac8, and Atg11 [120,121]. Furthermore, HTT plays an important role in autophagy by interacting with ULK1 and p62 [122] (Figure 3). HTT competes with mTOR for ULK1 and can induce autophagy by releasing ULK1 from mTORC1, which inhibits the activity of the ULK1/2 complex. HTT also binds to p62, which links ubiquitinated substrates and LC3 to bring them to an autophagosome. It has been shown that the deletion of polyQ in HTT enhances neuronal autophagy [123]. Thus, the conformational modification of HTT by polyQ expansion can restrain the autophagic pathways in the neurons and leads to neurodegeneration.

### 3.4. ALS

ALS is a fatal and progressive neurodegenerative disease that is caused by the degeneration of motor neurons. ALS is characterized by muscle weakness, atrophy, and paralysis. Approximately 90% of ALS cases are sporadic, and 10% of ALS cases are familial [124]. Although approximately 20 genes are associated with familial ALS, several familial ALS-associated genes, such as superoxide dismutase 1 (SOD1), C9orf72, TARDBP (TDP-43), and FUS, are associated with sporadic ALS in a small proportion of cases [125,126,127,128]. Furthermore, it has been reported that mutation in the Sigma 1 receptor (SigmaR1) is associated with autosomal recessive familial ALS [129]. The expression of SigmaR1 is high in motor neurons of the brain stem and spinal cord [130], and SigmaR1 knockout mice exhibit motor deficits with motor neuron degeneration [131]. SigmaR1 is a multifunctional protein involved in many cellular pathways [132] and is localized in the mitochondria-associated ER membranes (MAMs), where mitochondria interact with ER. SigmaR1 regulates calcium transport between mitochondria and ER at MAMs [133]. Knockdown of SigmaR1 leads to an increase in the release of calcium from ER, the depolarization of mitochondrial membrane potential, and apoptosis [134]. It also causes the inhibition of autophagic flux and autophagic degradation [135]. The prevention of autophagy leads to neurodegeneration [10,11]. These findings indicate that in ALS, the loss of function of SigmaR1 may lead to neurodegeneration in motor neurons. Recent exome sequencing studies found that TBK1 is an important protein for ALS [136,137]. TBK1 may be an important protein for both sporadic and familial ALS [136]. TBK1 phosphorylates OPTN, and phosphorylated OPTN plays an important role in mitophagy with PINK1 and Parkin by binding ubiquitinated OMM and LC3 [111,112] (Figure 2b). It has been reported that a mutation of OPTN is also present in ALS [138]. Moreover, a relationship between mitochondrial dysfunction and ALS has been reported [139,140]. Thus, the impairment of mitochondrial quality control by mitophagy may be an important factor for ALS as well as Parkinson’s disease.

### 3.5. SENDA

**Figure 4:**
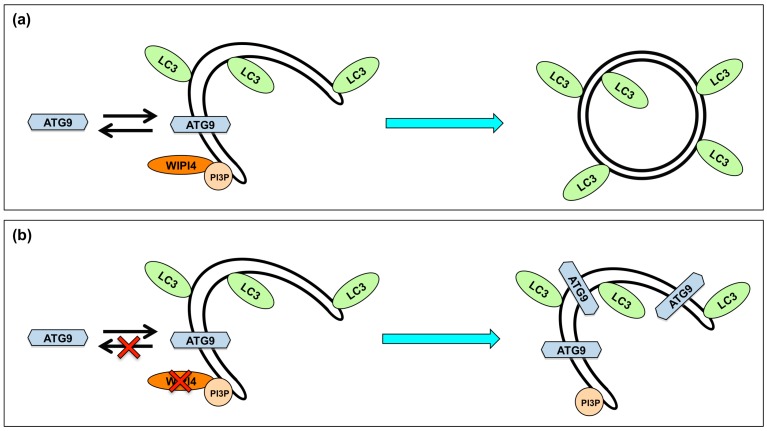
(**a**) WIPI4 may function to retrieve ATG9A from early autophagic vacuoles, and functional autophagosomes are formed with WIP4; and (**b**) the impairment of WIPI4 may lead to inhibition of the removal of ATG9A from early autophagic vacuoles and accumulation of early autophagic vacuoles. The red cross indicates loss of function. WIPI4: WD-repeat protein interacting with phosphoinositides 4.

SENDA is a neurodegenerative disease associated with the accumulation of iron in the brain. SENDA is characterized by global developmental delay in early childhood, and severe dystonia-parkinsonism and progressive dementia in adulthood [141]. Exome sequencing studies identified that WIPI4, which is encoded by the *WDR45* gene, is mutated in patients with SENDA [142,143]. WIPI4 is one of the WIPI proteins. WIPI proteins function to generate early autophagic vacuoles by being recruited to PI3P, which is generated by the Beclin1-VPS34 complex (Figure 1). The expression of WIPI4 and the autophagic activity were reduced in lymphoblastoid cell lines from patients with SENDA. The decreased expression of WIPI4 in patients with SENDA may be due to the structural instability of mutated WIPI4 and may result in the reduction of autophagic flux in patients with SENDA. Moreover, autophagic vacuoles with ATG9A were accumulated in lymphoblastoid cell lines in patients with SENDA [143]. This indicates the accumulation of early autophagic vacuoles because ATG9A associates only transiently during the autophagosome formation and is not present in the mature autophagosomes. WIPI2 is considered crucial for retrieving ATG9A from early autophagic vacuoles because its impairment elicits the accumulation of ATG9A in such vacuoles [144]. Similar to WIPI2, WIPI4 may also function to retrieve ATG9A from such vacuoles, and its impairment may also lead to inhibition of the removal of ATG9A from early autophagic vacuoles (Figure 4).

Further investigation is required to understand the detailed mechanism regarding the dysfunction of WIPI4 and the impairment of autophagy in patients with SENDA.

## 4. Concluding Remarks

In this review, we have summarized recent findings associated with the relationships between autophagy and neurodegenerative diseases. Autophagy functions to maintain cellular homeostasis and degrade aggregated proteins and dysfunctional organelles. The impairment of the autophagic process disturbs neuronal homeostasis and may lead to neurodegeneration because neurons are post-mitotic cells and it is difficult to dilute toxic proteins and dysfunctional organelles by mitosis. Thus, the elucidation of the mechanism of autophagy is indispensable to uncover the etiology of neurodegenerative diseases. Whole-exome sequencing can detect the difference of single base pairs in all exons of human genes, and could lead to the discovery of other mutated genes that are associated with neurodegenerative diseases. Thus, new technologies, such as whole-exome sequencing, will help unravel the complex role of autophagy in neurodegeneration. A better understanding of the detailed mechanisms of autophagic signaling pathways in the brain will contribute to the development of new therapeutic approaches for the improvement and prevention of neurodegenerative diseases.
